# Patterns of Quetelet Index (Body Mass Index) Improvement and Associated Clinical Factors During Initial Tuberculosis Treatment: A Prospective Analysis of Newly Diagnosed Tuberculosis Patients

**DOI:** 10.7759/cureus.80446

**Published:** 2025-03-12

**Authors:** Rock B Dharmaraj, Madhan Mohan, Neethu George, Vellaiappan P Hariharan, C. Brilly Swarna, Tamilarasan Muniyapillai, Neeraj V Mohandas, Vijay Anand V, Karthikeyan Kulothungan

**Affiliations:** 1 Community Medicine, Dhanalakshmi Srinivasan Medical College and Hospital, Perambalur, IND; 2 Respiratory Medicine, Dhanalakshmi Srinivasan Medical College and Hospital, Perambalur, IND

**Keywords:** anthropometry, body mass index, newly diagnosed tuberculosis, quetelet index, tuberculosis

## Abstract

Introduction

Tuberculosis (TB) and malnutrition form a detrimental synergistic relationship, with malnutrition weakening the immune system and increasing susceptibility to TB, while TB, in turn, worsens the nutritional status through increased metabolic demands and decreased appetite. The nutritional recovery is expected during treatment, but the extent and rate of improvement may vary based on patient characteristics, disease severity, and presence of comorbidities. This study aimed to assess the nutritional status changes in newly diagnosed TB patients by evaluating and comparing their body mass index (BMI) at treatment initiation and after the completion of intensive phase (two months) while also analysing the influence of various factors (age, gender, bacterial load, drug sensitivity, and comorbidities) on BMI variations during this period.

Methods

A prospective longitudinal study was conducted among 116 newly diagnosed pulmonary TB patients at a tertiary medical college hospital in Tamil Nadu between August and October 2021. Baseline assessments included demographic data, sputum microscopy for bacterial load, drug sensitivity testing via the Cartridge-Based Nucleic Acid Amplification Test (CBNAAT)/TrueNat, and screening for diabetes mellitus and chronic kidney disease (CKD). Anthropometric measurements (weight and BMI) were recorded at treatment initiation and after the completion of a two-month intensive phase treatment using standardized protocols. Data analysis was performed using IBM SPSS Statistics for Windows, V. 23.0 (IBM Corp., Armonk, NY, USA), with descriptive statistics for demographic and clinical characteristics, comparative analyses using independent t-test and one-way ANOVA for subgroup BMI improvements, and McNemar's test and paired t-test for assessing changes in BMI from baseline to post-two-month treatment (p < 0.05 considered significant).

Results

Among 116 pulmonary TB patients, mean (SD) BMI significantly increased from 16.17 (4.15) to 16.96 (4.01) kg/m² during the intensive phase treatment (p < 0.001). Of 89 initially malnourished patients, 9% achieved normal BMI status. Younger patients (<30 years) showed higher BMI improvement (1.12 ± 0.51 kg/m²) compared to the elderly (>70 years: 0.56 ± 0.49 kg/m²). Lower BMI improvements were observed in rifampicin-resistant cases (0.48 ± 0.22 vs 0.80 ± 0.50 kg/m²) and patients with comorbidities (diabetes: 0.74 ± 0.53 vs 0.85 ± 0.46 kg/m²; CKD: 0.65 ± 0.51 vs 0.83 ± 0.49 kg/m²), though these differences were not statistically significant.

Conclusion

Significant improvements in nutritional status were observed during the intensive phase treatment, with younger age associated with better BMI gains. Though not statistically significant, drug resistance and the presence of comorbidities appeared to negatively influence nutritional recovery, suggesting the need for enhanced nutritional monitoring and support in these vulnerable subgroups.

## Introduction

Tuberculosis (TB), caused by *Mycobacterium tuberculosis* (MTB), remains one of the most formidable public health challenges globally and continues to be the leading cause of mortality from a single infectious agent. The Global TB Report 2022 highlighted the magnitude of this challenge, documenting an estimated 10.6 million incident TB cases in 2021 [1]. Within the global TB landscape, India bears a disproportionate burden, accounting for 28% of worldwide cases among the six high-burden countries in the South-East Asia region [2]. The severity of India's TB epidemic is further underscored by the National TB Prevalence Survey 2021, which revealed a concerning crude prevalence of TB infection (TBI) of 31.3% among individuals aged 15 years and above [3].

The progression from TBI to active disease is influenced by a complex interplay of socioeconomic, environmental, and host-related factors. While poverty, poor housing conditions, and economic deprivation create the foundational context for TB transmission, the pathway to active disease is mediated through multiple risk factors. Initial infection risk is primarily driven by exogenous factors including exposure intensity, duration of contact, and environmental conditions such as overcrowding and poor ventilation. However, the progression to active TB disease is predominantly influenced by host-related factors that compromise immune function. These include malnutrition, human immunodeficiency virus (HIV) co-infection, diabetes, excessive alcohol consumption, tobacco use, and indoor air pollution exposure. While HIV co-infection remains the most potent individual risk factor, the population-level impact of factors such as malnutrition and diabetes may be more substantial in regions with a high prevalence of these conditions but lower HIV rates [4-9].

Malnutrition plays a pivotal dual role in TB, acting both as a risk factor for disease development and because of the disease process itself. Through its profound impact on cell-mediated immunity, the primary defense mechanism against TB, malnutrition significantly increases susceptibility to both primary TBI and the reactivation of latent TB. This relationship becomes particularly evident in the context of HIV co-infection, where nutritional deterioration often triggers TB reactivation. Conversely, active TB disease itself induces a state of malnutrition through increased metabolic demands, decreased appetite, and altered metabolism, creating a detrimental cycle that can worsen disease progression and clinical outcomes. This bidirectional relationship underscores the critical importance of nutritional assessment and intervention in TB management, as it directly influences disease progression, treatment response, and overall prognosis [6,7,10,11].

Body mass index (BMI) serves as a crucial anthropometric indicator in TB, reflecting both disease risk and treatment response. Low BMI increases TB susceptibility and predicts poorer outcomes, while TB itself induces weight loss through increased metabolism and reduced intake, creating a detrimental cycle. During treatment, monitoring BMI changes provides valuable insights into treatment effectiveness, with weight gain during the intensive phase often signalling favourable response. This bidirectional relationship between nutritional status and TB emphasizes the critical importance of regular anthropometric assessment in optimizing patient outcomes [12-19]. Many studies have demonstrated that a significant inverse relationship exists between BMI and TB, with reduced BMI not only increasing disease risk but also adversely affecting treatment outcomes, including six-month culture conversion rates and end-of-treatment results [20-22]. This study aimed to assess the nutritional status changes in newly diagnosed TB patients by evaluating and comparing their BMI at treatment initiation and after the completion of intensive phase (two months) while also analysing the influence of various factors (age, gender, bacterial load, drug sensitivity, and comorbidities) on BMI variations during this period.

## Materials and methods

Study design and setting

A prospective longitudinal study was conducted at Dhanalakshmi Srinivasan Medical College and Hospital, Perambalur, Tamil Nadu, between August and October 2021, involving both the Respiratory Medicine Outpatient Clinic (TB clinic) and Community Medicine Department.

Study population

A total of 116 newly diagnosed pulmonary TB patients came to the study setting during the study period and were enrolled by universal sampling. Patients were included if they had newly diagnosed active pulmonary TB confirmed by either three sputum specimens positive for acid-fast bacilli (AFB) (microscopy and culture), Cartridge-Based Nucleic Acid Amplification Test (CBNAAT)/TrueNat positivity, or clinical and radiological findings consistent with pulmonary TB. Those with previous anti-TB treatment were excluded. Baseline assessments included screening for diabetes mellitus (DM) (fasting serum glucose), chronic kidney disease (CKD) (serum creatinine), and rifampicin sensitivity.

Study procedure

In this longitudinal study, data collection was conducted by the primary investigator (student) with assistance from the surveillance officer at the TB clinic. Follow-up evaluations were systematically conducted at two-month intervals over the three-month study period. All participants were successfully contacted and evaluated during each scheduled follow-up visit, resulting in complete data sets with no attrition throughout the study duration. Prior to commencing the study, ethical approval was obtained from the Institutional Ethics Committee of Dhanalakshmi Srinivasan Medical College and Hospital (approval number: IECHS/IRCHS/DSMCH/41-2020). All participants provided informed consent, and the study was conducted in accordance with the Declaration of Helsinki guidelines for research involving human subjects.

Study tools

Patient Characteristics

Demographic data including age and gender were recorded using a structured proforma.

Bacteriological Assessment

Bacterial load was assessed through sputum microscopy using light-emitting diode (LED) fluorescence microscopy and graded according to WHO/Revised National Tuberculosis Control Programme (RNTCP) guidelines as scanty, 1+, 2+, and 3+ [23].

Drug Sensitivity Pattern

Drug sensitivity testing was performed using CBNAAT/TrueNat. Cases were classified as drug-sensitive, intermediate, or resistant based on these results [24].

Comorbidity Assessment

The presence of comorbidities such as DM (fasting blood sugar >126 mg/dl or postprandial blood sugar >200 mg/dl) and CKD (derangements in serum creatinine and glomerular filtration rate) was assessed.

Anthropometric Measurements

The following anthropometric measurements were obtained from all participants at two time points: treatment initiation (baseline) and after the completion of the two-month intensive phase treatment [25].

Body weight was measured using a calibrated electronic platform scale with a precision of 0.1 kg. Measurements were taken with participants wearing light clothing and no shoes and standing still in the centre of the scale platform with arms hanging freely by the sides and weight distributed evenly on both feet.

Height was measured to the nearest 0.1 cm using a wall-mounted stadiometer. Participants were measured standing barefoot, with heels together, arms at sides, legs straight, shoulders relaxed, and head in the Frankfort horizontal plane. The heels, buttocks, scapulae, and back of the head were positioned against the vertical board of the stadiometer.

BMI (Quetelet index) was calculated using the standard formula: weight (kg) divided by height squared (m²). BMI categories were defined according to WHO classification as follows: underweight: <18.49 kg/m²; normal weight: 18.5-24.9 kg/m²; overweight: 25-29.9 kg/m²; and obese: ≥30 kg/m² [26]. All measurements were performed by trained research staff following standardized protocols. To ensure measurement accuracy, the electronic scale was calibrated daily using standard weights. Each measurement was taken twice, and the average value was recorded. If the difference between two measurements exceeded preset tolerance limits (e.g., 0.1 kg for weight, 0.5 cm for height), a third measurement was taken, and the mean of the two closest values was used.

Statistical analysis

Statistical analysis was performed using appropriate statistical software (IBM SPSS Statistics for Windows, V. 23.0 (IBM Corp., Armonk, NY, USA)). Descriptive statistics were employed to summarize demographic and clinical characteristics, with frequencies and percentages calculated for categorical variables including gender, age groups, sputum AFB smear grades, molecular testing results, and comorbidities, while mean age was calculated for the study population. Comparative analyses were conducted to evaluate BMI improvement across different subgroups including age, gender, sputum smear status, DM and CKD status, and rifampicin sensitivity status using independent t-test and one-way ANOVA test. McNemar's test and paired t-test were utilized to assess the significance of changes in mean BMI and BMI categories (malnourished versus normal) from baseline to post two months of intensive phase treatment. Throughout the analysis, a p-value of <0.05 was considered statistically significant.

## Results

Age and sex distribution

Among the subjects studied, 92 (79.31%) were males and 24 (20.69%) were females. The mean age was 49.48 (5.76) years. In the study, 32 (27.59%) were in the 51-60-year-old age group, followed by 31 (26.72%) in the 41-50-year-old age group and at least seven (6.03%) in the >70-year-old age group. The age and sex distribution showed TB was more prevalent in males than females.

Confirmation of pulmonary TB and comorbid diseases

Analysis of sputum microscopy revealed that most patients had a high bacterial load, with 40 patients (34.48%) showing 2+ AFB smear positivity. Molecular diagnostic testing demonstrated that 106 patients (91.38%) were CBNAAT positive, with rifampicin sensitivity detected in 112 cases (96.55%). Regarding comorbidities, DM was present in 53 patients (45.69%), while CKD was documented in 20 patients (17.24%) (Table 1).

**Table 1:** Confirmation of pulmonary tuberculosis and comorbid diseases AFB: acid-fast bacilli; CBNAAT: Cartridge-Based Nucleic Acid Amplification Test; DM: diabetes mellitus; CKD: chronic kidney disease

	n	%
Sputum AFB smear		
1+	35	30.17
2+	40	34.48
3+	38	32.76
Negative	3	2.59
Molecular testing distribution-positive		
CBNAAT	106	91.38
TrueNat	10	8.62
Rifampicin sensitivity		
Intermediate	2	1.72
Resistant	2	1.72
Sensitive	112	96.56
Comorbid diseases		
DM present	53	45.69
DM absent	63	54.31
CKD present	20	17.24
CKD absent	96	82.76

BMI

The study population of 116 newly diagnosed pulmonary TB-positive patients were assessed before the initiation of treatment and after two months of intensive phase using their nutritional status. The mean BMI at presentation was 16.17 which is lower than the mean BMI after two months, which was 16.96, and the difference between BMI at presentation and BMI after two months was statistically significant (Table 2).

**Table 2:** Distribution of BMI at presentation and after two months of intensive phase *P-value < 0.05 is statistically significant; paired t-test BMI: body mass index; SD: standard deviation

	N		Mean	SD	Mean diff.	SD of diff.	P-value
Weight at presentation	116		46.86	11.54	-2.30	1.39	0.001*
Weight after two months	116		49.16	11.12			
BMI at presentation		116	16.17	4.15	-0.80	0.49	0.001*
BMI after two months		116	16.96	4.01			

Regarding the BMI at presentation of the subjects when compared with BMI after two months, out of 89 subjects who were initially underweight, eight (9%) improved to normal BMI after two months, and out of 27 individuals who initially had normal BMI, all maintained their nutritional status, and none of them degraded to underweight. The difference was statistically significant (p = 0.001) stating that the intervention of nutrition for two months had a significant effect on the subjects (Table 3).

**Table 3:** Distribution of BMI after two months with the BMI at presentation BMI: body mass index

BMI at presentation	BMI after two months		McNemar's test
	Underweight	Normal	
Malnourished	81 (91.01%)	8 (8.98%)	P-value: 0.001
Normal	0 (0%)	27 (100%)	

BMI improvement association with independent variables

Patients under 30 years demonstrated the highest mean BMI increase of 1.12 (SD ± 0.51), while those over 70 years showed the lowest improvement of 0.56 (SD ± 0.49). The middle age groups showed intermediate improvements ranging from 0.72 to 0.86 (p = 0.186). Regarding gender differences, female patients showed slightly better BMI improvement with a mean of 0.87 (SD ± 0.63) compared to males with 0.78 (SD ± 0.45) (p = 0.396).

The sputum AFB smear results, which indicate bacterial load, showed varied BMI improvements. Patients with 3+ and 1+ smears showed higher improvements (0.87 and 0.82, respectively) compared to those with negative or 2+ smears (both 0.71) (p = 0.495). Rifampicin-resistant cases showed lower BMI improvement (0.48, SD ± 0.22) compared to sensitive cases (0.80, SD ± 0.50), with intermediate resistance falling in between (0.64, SD ± 0.02) (p = 0.59) (Table 4).

**Table 4:** BMI improvement in two months stratified with independent variables BMI: body mass index; AFB: acid-fast bacilli; SD: standard deviation

		BMI improvement		P-value
		Mean	SD	
Age group	<30 years	1.12	0.51	0.186
	31-40 years	0.86	0.48	
	41-50 years	0.72	0.43	
	51-60 years	0.78	0.57	
	61-70 years	0.78	0.37	
	>70 years	0.56	0.49	
Sex	Males	0.78	0.45	0.396
	Females	0.87	0.63	
Sputum AFB smear	Negative	0.71	0.63	0.495
	1+	0.82	0.44	
	2+	0.71	0.41	
	3+	0.87	0.60	
Rifampicin sensitivity	Resistant	0.48	0.22	0.59
	Intermediate	0.64	0.02	
	Sensitive	0.80	0.50	

Comorbid diseases

The data presents BMI improvements stratified by the presence of comorbidities, specifically DM and CKD. For patients with DM, the mean BMI improvement was 0.74 kg/m² (SD ± 0.53), while those without DM showed a slightly higher mean improvement of 0.85 kg/m² (SD ± 0.46). The BMI changes ranged from a decrease of 0.33 kg/m² to an increase of 1.98 kg/m² in diabetic patients, compared to a range of -0.38 to 2.22 kg/m² in non-diabetic patients. However, this difference was not statistically significant (p = 0.244). Similarly, patients with CKD demonstrated a mean BMI improvement of 0.65 kg/m² (SD ± 0.51), while those without CKD showed a better improvement of 0.83 kg/m² (SD ± 0.49). The BMI changes in CKD patients ranged from no change (0.00) to an increase of 1.75 kg/m², whereas non-CKD patients showed changes ranging from -0.38 to 2.22 kg/m² (p = 0.155) (Table 5). 

**Table 5:** BMI improvement in two months stratified with the presence or absence of comorbid disease DM: diabetes mellitus; CKD: chronic kidney disease; BMI: body mass index; SD: standard deviation

		BMI improvement				P-value
		Mean	SD	Minimum	Maximum	
DM	Yes	0.74	0.53	-0.33	1.98	0.244
	No	0.85	0.46	-0.38	2.22	
CKD	Yes	0.65	0.51	0.00	1.75	0.155
	No	0.83	0.49	-0.38	2.22	

## Discussion

In this prospective study of 116 newly diagnosed pulmonary TB patients, we observed significant improvements in nutritional status during the intensive phase of treatment. The mean BMI increased from 16.17 kg/m² at baseline to 16.96 kg/m² after two months of treatment, with the improvement being statistically significant. Among the 89 initially malnourished patients, 9% improved to normal BMI status, while all 27 patients with normal baseline BMI maintained their nutritional status (p < 0.05).

A study done in South India [27] demonstrated a similar trend in weight changes during the intensive phase treatment. While this study focused on BMI changes, their cohort of 726 patients showed that 79.7% experienced weight gain during the intensive phase, with a mean gain of 2.2 kg among those who improved. Their study reported a notable finding that 46.4% of patients achieved >5% weight gain, though 18.7% experienced weight loss. The range of weight changes in their cohort was quite wide, spanning from a loss of 22.2 kg to a gain of 22.3 kg. This variability in weight changes aligns with our observations of differential BMI improvements across various patient subgroups, though our study additionally provides insights into specific factors such as age, drug resistance patterns, and comorbidities that might influence these nutritional outcomes. Their finding of 59.6% participants being underweight at baseline complements our observation of 76.7% (89/116) malnourished patients at treatment initiation, suggesting that malnutrition is indeed a significant concern in TB patients across different Indian settings.

The study demonstrated a significant mean weight gain of 2.30 kg (from 46.86 kg to 49.16 kg) during the intensive phase of treatment (p = 0.001). These findings can be compared with a retrospective cohort study from Peru [28] that provided outcome-stratified weight gain patterns. In their cohort, patients with good treatment outcomes gained approximately 1 kg after the first month, progressing to 3 kg by the fourth month, while those with poor outcomes showed weight loss of about 1 kg in the first month and minimal recovery (0.2 kg gain) by four months. The present cohort's mean weight gain of 2.30 kg at two months appears to align with the trajectory of their "good outcome" group, suggesting a favourable treatment response in our population. However, unlike the Peruvian study which provided outcome-stratified data, our findings represent aggregate weight changes across all patients, potentially including both those with excellent and poor recovery patterns. Nevertheless, the significant weight gain observed in our cohort during the intensive phase underscores the importance of nutritional recovery as a potential early indicator of treatment response. Similarly, a study from Tiruvallur district, Tamil Nadu [29], revealed substantial variations in weight changes during treatment, ranging from a loss of 4 kg to a gain of 20 kg at treatment completion. Another study from Malaysia [30] showed that 90% of the subjects had weight gain during the course of one month of treatment. While the present study focused on the intensive phase showing consistent improvement (mean gain of 2.30 kg), the Tiruvallur study demonstrates the potential for more substantial weight gains over the complete treatment duration. This observation is further supported by findings from Vietnam [31] and the Tuberculosis Trials Consortium Study [32], where patients who failed to gain weight or experienced weight loss during the initial two months of treatment were found to have unfavourable treatment outcomes or at relapse risk. A study conducted in the USA [33] demonstrated significant changes in patients' nutritional status during treatment, with mean BMI increasing from 23.2 ± 0.5 to 23.7 ± 0.6 kg/m² and mean weight improving from 63.9 ± 1.4 to 65.1 ± 1.7 kg.

A study from Guinea [34] focusing on multidrug-resistant (MDR) TB patients reported a monthly BMI increase of 0.24 kg/m² (SE 0.02). The study observation of lower BMI improvement in rifampicin-resistant cases (0.48 ± 0.22 kg/m²) compared to drug-sensitive cases (0.80 ± 0.50 kg/m²) aligns with these findings from Guinea, suggesting that drug resistance may influence the rate of nutritional recovery during treatment. A study conducted among drug-resistant cases in Lesotho [21] further reinforced this relationship, demonstrating that poor BMI changes were associated with unfavourable treatment outcomes and delayed sputum conversion. Additionally, a Chinese study [20] suggested that both underweight and overweight had been associated with multidrug resistance or single drug resistance in comparison to normal weight subjects. The USA study's [33] observation of the potential association between MDR-TB and weight loss adds to the growing evidence of compromised nutritional recovery in drug-resistant cases. Also, studies done in Indonesia [35] and the Philippines [36] showed that weight gain is a significant factor in determining treatment outcomes in drug-resistant TB. This finding complements the understanding of the complex relationship between drug resistance and nutritional status, as previously noted in our analysis where rifampicin-resistant cases showed lower BMI improvement compared to drug-sensitive cases. The emerging pattern suggests that drug resistance might not only affect treatment outcomes but also impair nutritional recovery, possibly due to prolonged illness, more aggressive treatment regimens, and increased metabolic stress. These collective findings underscore the complex bidirectional relationship between nutritional status and drug resistance in TB, highlighting the need for targeted nutritional interventions and the careful monitoring of BMI throughout the treatment course, particularly in drug-resistant cases.

In a study conducted in Guinea [34], females showed a significantly slower BMI increase (68.2% vs 27.3%), whereas in this study, females showed marginally better BMI improvements (0.87 ± 0.63 kg/m²) than males (0.78 ± 0.45 kg/m²; p = 0.396). This divergence could be attributed to differences in study population characteristics and socioeconomic conditions between the two settings. Notably, the study findings showing marginally better BMI improvements in females did not reach statistical significance (p = 0.396), suggesting the observed gender difference might be due to chance. A Tanzanian study [37] showed that mean weight gain was similar between sexes, but males had higher fat-free mass gain in comparison with females.

The observed relationship between age and weight gain during TB treatment aligns with findings from the USA study [33], which demonstrated that patients over 60 years of age gained less weight compared to those younger than 60 years (p = 0.04). This study similarly found that younger patients under 30 years showed the highest mean BMI increase (1.12 ± 0.51 kg/m²) compared to elderly patients over 70 years (0.56 ± 0.49 kg/m²), though this difference did not reach statistical significance (p = 0.186). This consistent pattern across different populations suggests that age is an important determinant of nutritional recovery during TB treatment.

In this study, the presence of comorbidities appeared to influence nutritional recovery, with DM and CKD patients showing relatively lower BMI improvements (DM: 0.74 ± 0.53 vs 0.85 ± 0.46 kg/m²; CKD: 0.65 ± 0.51 vs 0.83 ± 0.49 kg/m²) compared to those without these conditions, though these differences were not statistically significant (p = 0.244 for DM and p = 0.155 for CKD). These findings align with research from the USA [33], where certain comorbidities like DM, specifically malignancy and hepatitis B/C co-infection, were identified as significant negative predictors of percentage weight gain during treatment. The consistency of these observations across different settings and comorbidity types suggests that concurrent medical conditions may impair nutritional recovery during TB treatment, possibly due to altered metabolism, increased inflammatory burden, or medication interactions.

The heterogeneity in weight and BMI gains with associated factors during TB treatment across global studies can be attributed to several key factors. Host-related variables including age, genetic background, and baseline nutritional status influence recovery patterns, while comorbidities like DM and kidney disease may impair improvement through metabolic alterations. Disease-specific factors, particularly drug resistance, affect treatment efficacy and subsequent nutritional recovery. Regional variations in dietary practices and nutritional support programs, combined with healthcare system differences in monitoring and adherence support, contribute to these disparities. Additionally, socioeconomic factors affecting access to adequate nutrition play a crucial role in determining recovery patterns.

Limitations

The relatively small sample size of 116 patients and the single-centre design may limit the generalizability of the findings. The two-month follow-up period, while covering the intensive phase, doesn't capture the complete trajectory of nutritional recovery throughout the full treatment course. The study was unable to assess important confounding factors such as socioeconomic status, dietary intake patterns, and physical activity levels which could influence nutritional recovery. Additionally, the use of BMI alone as a nutritional indicator, without other anthropometric measurements or biochemical markers, may not provide a complete picture of nutritional status.

Strengths

The study's prospective design with standardized measurements at baseline and follow-up represents a key strength, minimizing recall bias and ensuring data quality. The comprehensive analysis of multiple factors including age, gender, drug resistance patterns, and comorbidities provides valuable insights into various determinants of nutritional recovery. The inclusion of both drug-sensitive and drug-resistant cases, along with patients across different age groups and comorbidity status, offers a realistic representation of the typical patient population. Furthermore, the timing of the assessment during the crucial intensive phase provides important early indicators of treatment response.

Recommendation

Based on the findings, strengthening of the existing nutritional support frameworks within India's TB control program should be done. The Nikshay Poshan Yojana, which provides monthly financial support for nutritional needs, should be efficiently implemented with timely disbursement of funds and focus on community engagement and nutritional awareness. Also, routine nutritional monitoring with standardized protocols during TB treatment should be implemented, with particular attention to vulnerable subgroups such as elderly patients and those with comorbidities. Strengthening the convergence with other nutrition-focused schemes like Anganwadi services and mid-day meal programs could provide additional nutritional support to TB patients. Future research should include larger, multi-centre studies with longer follow-up periods to better understand the complete trajectory of nutritional recovery. Integration of additional nutritional assessment tools, including body composition analysis and biochemical markers, would provide a more comprehensive evaluation.

## Conclusions

The intensive phase of TB treatment demonstrates significant improvement in the anthropometric status of newly diagnosed pulmonary TB patients, evidenced by a statistically significant increase in mean BMI. While most initially malnourished patients showed BMI improvement, and all patients with normal baseline BMI maintained their nutritional status, this improvement varied across different patient subgroups. Although age, gender, drug sensitivity, and presence of comorbidities appeared to influence the degree of nutritional recovery, these differences were not statistically significant.
